# Impacts of reopening strategies for COVID-19 epidemic: a modeling study in Piedmont region

**DOI:** 10.1186/s12879-020-05490-w

**Published:** 2020-10-28

**Authors:** Simone Pernice, Paolo Castagno, Linda Marcotulli, Milena Maria Maule, Lorenzo Richiardi, Giovenale Moirano, Matteo Sereno, Francesca Cordero, Marco Beccuti

**Affiliations:** 1 Department of Computer Science, University of Torino, Corso Svizzera 185, Torino, 10149 Italy; 2 Cancer Epidemiology Unit, Department of Medical Sciences, University of Torino - CPO Piemonte, Via Santena 7, Torino, 10126 Italy

**Keywords:** COVID-19, Mechanistic models, Control strategies

## Abstract

**Background:**

Severe acute respiratory syndrome coronavirus 2 (SARS-COV-2), the causative agent of the coronavirus disease 19 (COVID-19), is a highly transmittable virus. Since the first person-to-person transmission of SARS-CoV-2 was reported in Italy on February 21^st^, 2020, the number of people infected with SARS-COV-2 increased rapidly, mainly in northern Italian regions, including Piedmont. A strict lockdown was imposed on March 21^st^ until May 4^th^ when a gradual relaxation of the restrictions started. In this context, computational models and computer simulations are one of the available research tools that epidemiologists can exploit to understand the spread of the diseases and to evaluate social measures to counteract, mitigate or delay the spread of the epidemic.

**Methods:**

This study presents an extended version of the Susceptible-Exposed-Infected-Removed-Susceptible (SEIRS) model accounting for population age structure. The infectious population is divided into three sub-groups: *(i)* undetected infected individuals, *(ii)* quarantined infected individuals and *(iii)* hospitalized infected individuals. Moreover, the strength of the government restriction measures and the related population response to these are explicitly represented in the model.

**Results:**

The proposed model allows us to investigate different scenarios of the COVID-19 spread in Piedmont and the implementation of different infection-control measures and testing approaches. The results show that the implemented control measures have proven effective in containing the epidemic, mitigating the potential dangerous impact of a large proportion of undetected cases. We also forecast the optimal combination of individual-level measures and community surveillance to contain the new wave of COVID-19 spread after the re-opening work and social activities.

**Conclusions:**

Our model is an effective tool useful to investigate different scenarios and to inform policy makers about the potential impact of different control strategies. This will be crucial in the upcoming months, when very critical decisions about easing control measures will need to be taken.

## Background

Italy was the first European country affected by the coronavirus 2 (SARS-CoV-2) outbreak, with the first autochthonous case identified in Lombardy on February, 21^st^, 2020 [1]. During the following weeks the number of people who tested positive for SARS-CoV-2 swab rapidly increased, exceeding 100,000 cases by the end of March 2020 [2, 3].

Undetected infections, being generally characterized by mild or no symptoms, can expose a large portion of the population to the virus and play a relevant role in the SARS-COV2 transmission. To reduce the spread of COVID-19, the Italian government introduced different restrictions, starting in the northern regions, where the first cases were detected, and then in the entire country. The first line of control was addressed to the closure of schools and museums. Later, people were encouraged to start smart working, and all the sports events were performed behind closed doors (February, 25^th^). The second intervention was focused on the closure of all the public activities involving crowd of people, restaurants and commercial activities; moreover, it was forbidden to cross the municipal borders (March, 8^th^). Finally, the latest control strategy imposed the total lockdown of the country halting non-essential production, industries and businesses (March, 21^st^). In the weeks following the third restriction, a slow but constant decrease of the infected cases was registered showing that the adopted control strategies had been effective in limiting the outbreak progression.

Starting from May 4^th^, these restrictions were gradually relaxed by the Italian government. In particular, work activities as manufacturing and wholesale were re-activated, and outdoor activities and the movements within each region boundaries were permitted. A complete reactivation of all the work activities was planned for the first week of June, while the school re-opening was postponed to September. At the same time, the government has required the intensification of infection-control measures (i.e., mask, gloves, social distancing), including specific rules to be adopted in workplaces, public places and transportation. The potential of tracing the cases’ contacts and testing was also increased. In these contexts, computational models can be very helpful for evaluating COVID-19 epidemic evolution, and the effects of different infection-control strategies such as human interaction controls, and other social measures that can impact on disease spreading dynamics.

Several models, often with conflicting results [4], have been proposed to investigate the COVID-19 pandemic. Models can be roughly classified as phenomenological and mechanistic. The former [5, 6] are formulated with the main aim of describing the epidemic pattern and make short-term predictions. The latter, such as the one we are proposing here, model the disease spread under various assumptions about the transmission process and the human and social contexts of the epidemic. These are used to obtain long-term forecasts and, possibly more important, to simulate different scenarios modulating the parameters that characterize variations in disease features and control measures. The model proposed by Ferguson and colleagues [7] had a strong impact in shaping the policies of several European countries and the US. In another influential model, Kissler and colleagues [8] explored the dynamics of COVID-19 over a period of several years, raising the possibility that repeated lockdowns may be necessary to keep levels of COVID-19 hospitalisations and deaths to manageable levels. Specific models have been proposed to describe the Italian epidemic development. The modelling study proposed by the Imperial College team [9] evaluates different scenarios for a relaxation of isolation measures, using increase in mobility as a proxy, and attempts to predict the *second wave* in terms of infection and death excesses. Another model, proposed by Giordano and colleagues [10] takes into account the distinction between diagnosed and non-diagnosed cases and points to the necessity of combining social-distancing measures with widespread testing and contact tracing to control the epidemic.

In this study, we propose an extended version of the Susceptible-Exposed-Infected-Removed-Susceptible (SEIRS) model to investigate COVID-19 spread disease. In particular, the novelties and strengths of this model can be summarized as follows: (i) the division of the infected subjects in three categories: undetected, quarantined, and hospitalized; (ii) the explicit representation of the population age structure; (iii) the usage of age-specific and location-specific contact matrices; (iv) the modeling of the government actions and the corresponding population response depending on the public perception of the disease severity; (v) the modeling of different infection-control measures, including individual-level measures, whose efficacy is subjected to the public perception of current disease severity, and SARS-CoV-2 swab testing capability.

Our model is then used to investigate different scenarios of COVID-19 diffusion in the Piedmont region by taking into account for the next three months following the gradual re-opening of May 4^th^. In particular, we studied how the COVID-19 spread in Piedmont could be kept under control by the implementation of the infection-control measures based on the use of individual-level measures (i.e., mask, gloves and social distancing), and on the intensification of the surveillance methods including contact tracing, the identification of undetected cases by swab testing, and early isolation of infected individuals. In conclusion, our model introduces important novelties in the modelling strategies used to investigate the COVID-19 outbreak, and may be used to support government decision-makers.

## Methods

This section is divided into two paragraphs describing the software and hardware exploited through the analysis and an exhaustive description of the model.

### Software and hardware used for the study

All the reported experiments were performed on a server with 6 Intel Xeon E5-2650 processors (2.00Ghz, 20MB Cache, 8 Cores) using *Epimod* [11] (https://github.com/qBioTurin/epimod), a tool recently developed by our group to provide a general framework to draw and analyze epidemiological systems. Epimod provides an easy but powerful environment whose strengths are: (1) the use of a graphical formalism to simplify the model creation phase; (2) the implementation of an R package providing a friendly interface to access the analysis techniques implemented in the framework; (3) a high level of portability and reproducibility granted by the containerization of all analysis techniques implemented in the framework; (4) a well-defined schema and related infrastructure to allow users to easily integrate their own analysis workflow in the framework.

### Model description

**Fig. 1:**
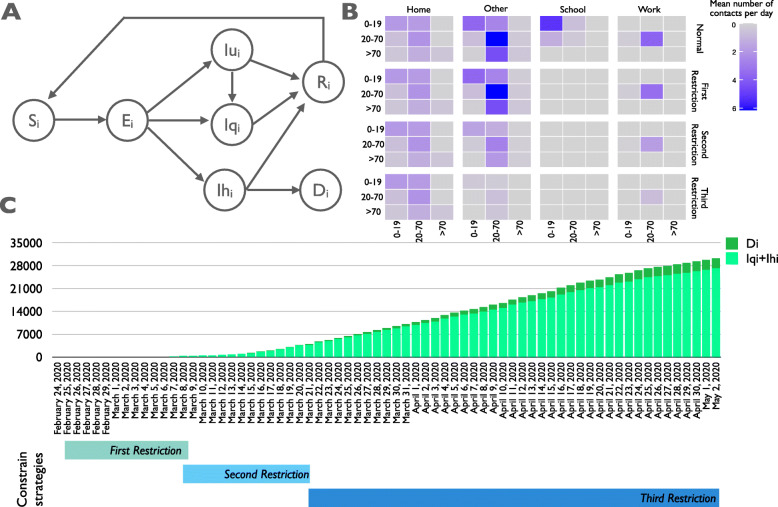
SEIRS model and surveillance data on Piedmont region. **a** The transmission flow diagram of our age-dependent SEIRS model. **b** Age-specific and location-specific contact matrices. The intense of the color indicates higher propensity of making the contact. **c** Distribution of infected cases as sum of quarantined (*I*_*qi*_) and hospitalized (*I*_*hi*_) infected (light green) and deaths (*D*_*i*_) (dark green) from February 24th to May 2nd. The periods of the activation of the three control strategies are reported below the stacked bars plot

We propose an extended version of the SEIRS model to account for the population age distribution, that was classified into three groups: young individuals 0-19 years, adults 20-69 years, old adults aged at least 70 years. The corresponding transmission flow diagram for a specific age class *i* is shown in Fig. 1a, where the circles represent population partitions and the arcs describe the disease progression.

The population of the age class *i* is partitioned in the following seven compartments: *susceptible* (*S*_*i*_), *exposed* (*E*_*i*_), *undetected infected*(*I*_*ui*_), *quarantined infected* (*I*_*qi*_), *hospitalized infected* (*I*_*hi*_), *recovered* (*R*_*i*_), *dead* (*D*_*i*_). The partition of the infectious population allows us to model quarantine practices and the effects of government control strategies specifically for each sub-class of individuals. Similarly, the division of population in three-age groups allows us to define age-dependent rates for the system events (e.g., infection, recovery, …, see Supplementary Material S1). We can thus model a scenario in which younger individuals, known to be more often asymptomatic or pauci-symptomatic, are more likely undetected than the older ones.

With respect to the classical SEIRS model, we have added a transition from *I*_*ui*_ to *I*_*qi*_ to model the possibility to identify undetected cases and isolate them. In this way an individual in *I*_*ui*_ tested as positive to the SARS-CoV-2 swab will be moved in the quarantine regime, *I*_*qi*_. This feature is crucial to capture the time varying diagnostic ability throughout the epidemic evolution, as shown by the increasing number of tests performed [12], as well as to forecast the effect of enhanced or decreased testing capabilities.

The social mixing pattern in the population is described by the age-specific and location-specific contacts depicted in the matrices reported in Fig. 1b. Social contacts change across contexts (i.e. home, work, school and other locations) and age-groups [13]. The effect of the public restrictions imposed by the Italian government was simulated by reducing social mixing contacts in all categories.

The *force of infection (FOI)* adopted in the model is a time and age class dependent function and includes the following four terms:

- the *infection rate*, depending on the age classes of both the susceptible and the infected individuals who come into contact according to the contact matrix;
- the *strength of governmental restriction* defined through a time-depended step function, modeling the severity of the public restrictions;
- the *compliance with the governmental restriction*, reporting how effectively the population adheres to the restriction measures imposed by the Italian government. The higher the disease severity (i.e., the severity of the epidemic in terms of number of deaths and hospitalized individuals in the last 40 days), the better the population compliance [14];
- the *compliance with individual-level measures*, considering how different infection-control measures are properly adopted by the population.

All demographic changes in the population (i.e., births, deaths, and ageing) are explicitly disregarded in our model as negligible due to the short time interval considered in our study. A detailed description of the model is reported in the Supplementary Material S2.

## Results

### Model calibration

The surveillance Piedmont data available at the website of the Italian Ministry of Health / Civil Protection [12] were used to calibrate our model. Among the data, the surveillance report publishes three categories of monitored individuals: quarantined infected (*I*_*qi*_), hospitalized infected (*I*_*hi*_) and deceased (*D*_*i*_), whose cumulative trends are reported in Fig. 1c. In the same Figure, the time points at which the control strategies were imposed are also shown.

The calibration phase was performed to fit the model outcome with the infection (*I*_*qi*_ + *I*_*hi*_) and death (*D*_*i*_) data from February 24^th^ to May 2^nd^ using squared error estimator via trajectory matching. In this phase, the model calibration was carried out considering the proportion between undetected and detected infected individuals (i.e., given by the sum of the quarantined and hospitalized infected individuals) to be one-to-one on average (as reported in [15]). Moreover, the average immunization period of recovered individuals was set to one year and half (see Supplementary Material Table S1).

As since April 1^st^ a tangible increment of the SARS-CoV-2 swab tests in long-stay residential care homes was implemented in Piedmont [16], we explicitly modeled the diagnostic ability to identify undetected cases among the old adults (70+) starting from the beginning of April.

**Fig. 2:**
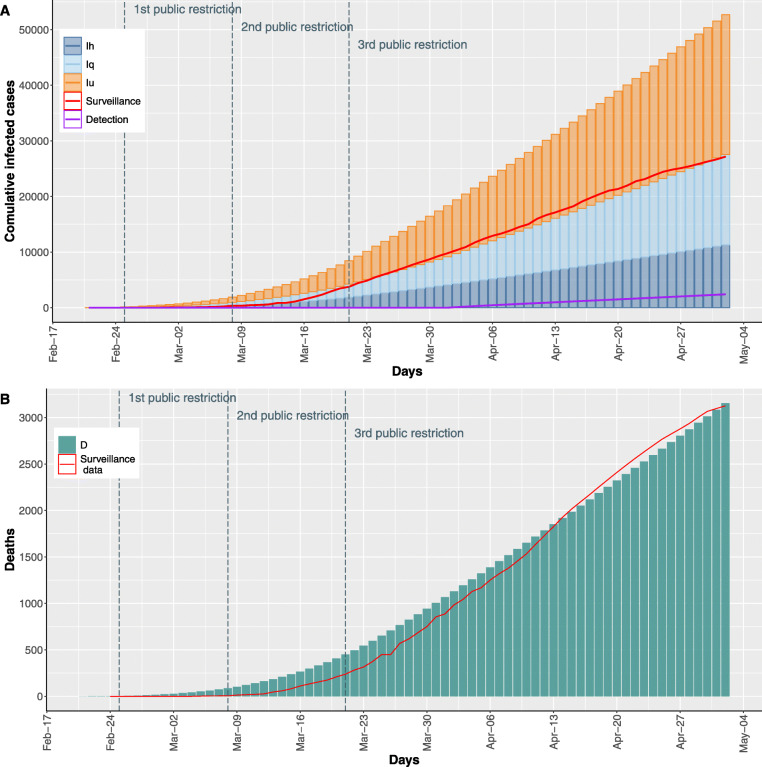
**a** Stacked bars plot reports the cumulative trend of the infected individuals in which the undetected infected are showed in orange, the quarantine infected in light blue, and hospitalized infected in blue. The purple line reports the cumulative trend of the undetected cases diagnosed by SARS-CoV-2 swab tests. **b** Histogram shows the cumulative trend of deaths. In both histograms the surveillance data are reported as red line

The plots in Fig. 2a and b show the time evolution of infected and deceased individuals derived by the model considering the optimal parameter values estimated by the calibration phase (Table S3 in the Supplementary Material). In details, the stacked bar chart in Fig. 2a shows the proportion of infected individuals in each of the three infectious sub-classes considered. The red line reports the detected infected individuals derived from the surveillance data, while the purple line reports the number of cases diagnosed in the elderlies that would have been undetected had the testing procedures not changed since April 1^st^.

Figure 2a reveals a good level of accordance between the infected individuals derived by the surveillance data and those derived by the calibrated model (i.e., given by the sum of the *I*_*qi*_ reported with light blue bars and *I*_*hi*_ reported by blue bars).

Consistently, Fig. 2b shows that the calibrated model is able to mimic consistently the observed death cases (red line). The plots reporting infected and deceased individuals for each age class in the same time interval are shown in Figures S2 and S3 in the Supplementary Material.

### The COVID-19 spread and the government control interventions

To study how the government control interventions and the corresponding population response affected COVID-19 diffusion, we focused on the third restriction, when a strict lockdown was enforced in Italy between from March 21^*st*^ to May 1^*st*^. In particular, we used our model to compare the infection spread under the following three scenarios: (i) the third restriction is activated from March 21^*st*^ and the population response is estimated by the surveillance data; (ii) the model extends the second restriction beyond March, 21^*st*^ without implementing the third restriction and the population response is the one estimated from surveillance data; and (iii) the third restriction is activated from March 21^*st*^ and the population response is higher than the one estimated by the surveillance data.

**Fig. 3:**
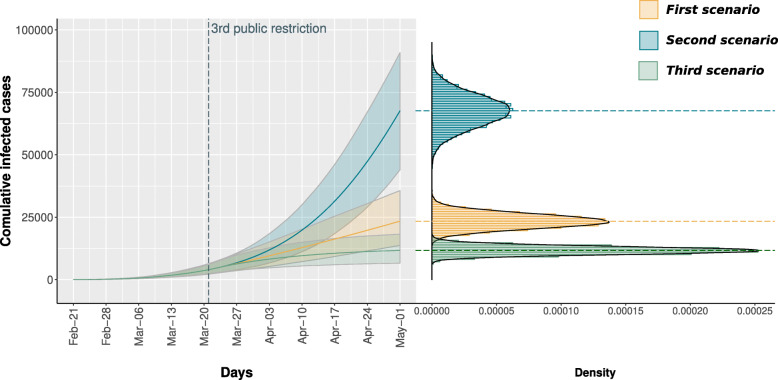
Stochastic simulation results reported as traces (on the left) and as density distributions (on the right). Three scenarios are implemented. In the *First scenario* the model is calibrated to fit the surveillance data (yellow). In the *Second scenario* the model extends the second restriction beyond March, 21^*st*^ without implementing the third restriction (blue). In the *Third scenario* the model consider a higher population compliance to the third governmental restriction (green)

Figure 3 shows the stochastic simulation traces (on the left) and the density distributions on May 1^*th*^ (on the right) of the total number of detected infected individuals, considering the three scenarios proposed above: yellow, blue and green for the first, second and third scenario, respectively. For each scenario 10’000 traces are simulated and the corresponding median trace is reported as a bold line. It is possible to appreciate that the third restriction was effective in containing the spread of the virus. In particular, the distribution under the first scenario, representing the observed data, is much closer to the third scenario, in which an almost complete compliance with the restriction is simulated, than to the second scenario assuming no lockdown and yielding a dramatically higher number of cases.

### COVID-19 epidemic containment strategies

**Fig. 4:**
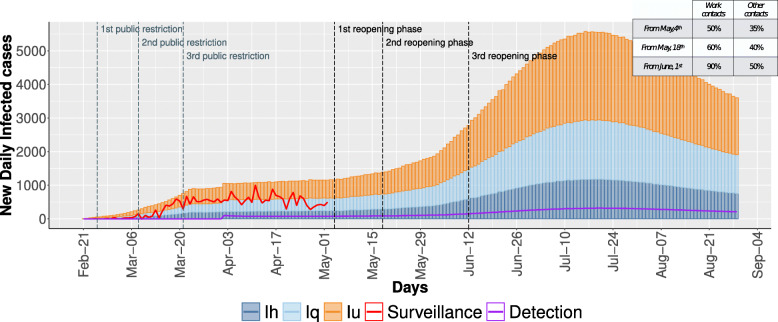
The daily evolution of infected individuals computed by the stochastic simulation. The stacked bars report the undetected infected (orange), the quarantine infected (light blue), and hospitalized infected (blue). The red line shows the trend of the infected cases from surveillance data. The purple line reports the cumulative trend of the undetected cases diagnosed by SARS-CoV-2 swab tests

From May, 4^*th*^ the restrictions imposed by the Italian government were gradually relaxed: the roadmap for lifting COVID-19 restrictions defined by the Italian Government sets out three reopening phases that are depicted in Fig. 4. In this context, our model can be effectively used to forecast the daily trend of the infected individuals until September 1^*st*^ considering this progressive increment of the social mixing patterns and implementing different infection-control measures.

Figure 4 shows a pessimistic scenario in which the gradual reopening is not counterbalanced by any infection-control strategies. The stacked bars report the predicted infected cases (blue and light blue) and the number of undetected individuals (orange), whereas the red line shows the surveillance data until May 1^*st*^. Moreover, the purple line reports the daily trend of otherwise undetected cases diagnosed by SARS-CoV-2 swab tests in the model.

After a first constant increment of the infected individuals in February and March, a plateau was reached from April, 3^rd^ to May, 1^*st*^. From that moment, the gradual re-opening of the working activities would cause a new increment of infected individuals, reaching a peak of about 7,000 daily new infected cases on July, 20^*th*^ when a gradual decrease would be produced by the population response to the severity of the epidemic. Starting from this worst-case scenario, we analyzed the cost-benefit trade-off between the implementation of infection-control measures and the relaxation of public restrictions. In particular, we consider 15 different scenarios arising from the combination of different levels of implementation/efficacy of two control measures: (i) use of individual-level measures, and (ii) increased case detection by contact tracing, swab testing and early quarantine regime of identified cases.

**Fig. 5:**
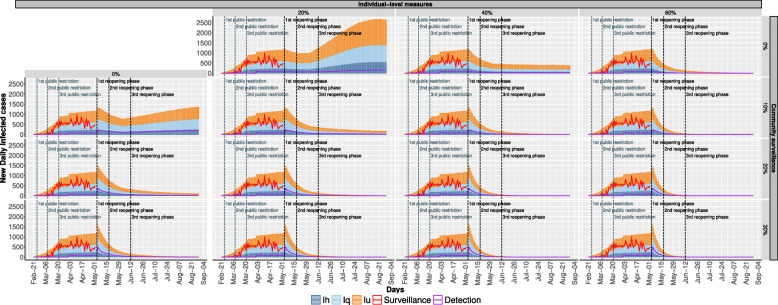
The daily evolution of infected individuals is shown varying on the columns the efficacy of individual-level measures and on the rows the efficacy of community surveillance

Specifically, in Fig. 5 we show the daily forecasts of the number of infected individuals with the efficacy of individual-level measures ranging from 0% to 60% on the columns (increasing by steps of 20%) and, on the rows, increasing capability (from 0% to 30%, by 10% steps) of identifying otherwise undetected infected individuals. These results are obtained as median value of 5000 traces for each scenario obtained from the stochastic simulation.

Comparing results in Fig. 5 with the pessimistic scenario of Fig. 4 it is important to notice that, regardless the efficacy combination we pick, the employment of infection-control strategy always exhibits a positive effect on the number of infected individuals, either flattening the peak or the number of infected individuals toward zero. Furthermore, the proposed set of scenarios shows that the combination of the two infection-control measures leads to envisage reasonable levels of protection arising from the adoption of individual-level measures (in the range of 20-40%) and the necessity to identify a feasible fraction of undetected cases. It is important to notice that the fraction of revealed undetected cases is not the reference measure for the number of people to test for Sars-CoV-2 infection. This rather represents the fraction of all undetected patient that, thanks to an enhanced testing approach, are eventually tested and identified. To reach this goal, the actual number of swabs to perform depends on the positive predictive value of the test, which, in turns, depends on the prevalence of SARS-CoV-2 positive individuals in the tested population. Thus, the same result can be achieved with a thorough contact tracing and targeted testing in high risk groups or with a larger number of untargeted (or less targeted) tests. The former approach is more efficient and feasible.

## Discussion

COVID-19 is an infectious disease transmissible via direct contact among individuals. This implies that governmental actions to limit physical proximity and interactions in the population are crucial to counteract the infection outbreak These actions become even more significant when the diffusion of the pathogen relies on a pool of undetected cases not showing symptoms.

In this study, we employed a multi-group SEIRS model as a basic modelling tool. We considered an age-structured population. Differentiating age classes allows us to mimic the real incidence of COVID-19 both for infections and fatalities. We also introduced a further layer to characterize categories of infected, so that also the severity of the infection and its associated quarantine regime can be modelled. The multi-group nature of our model allows us to investigate the peculiarities of the COVID-19 transmission, including different incidences or symptoms severity in different age classes. In our model, the time evolution of the infection is affected by governmental policies and the associated population awareness. Given the complex dynamics driving the behaviour of the population, we further modelled the perception of the hazard of COVID-19, relating it with the number of fatalities.

Firstly, we focused on the impact of the control strategies to limit contacts in the population. We modelled the impact of the control strategies by modulating the parameters that modify the strength of the governmental restriction and the population compliance with these restriction. Intervention scenarios that intensify the limitation of the person-to-person interactions correspond to an increase in these terms. We investigated the impact of the third public government restrictions on the evolution of the COVID-19 epidemic in Piedmont and found that the strict national lockdown and the related population response had a strong impact on the epidemic control. Model outcomes clearly highlight that the implementation of the sole second restriction would have not been enough to counteract the outbreak.

Secondly, we pinpointed the optimal combination of containment strategies based on individual-level measures and community surveillance to cope with the COVID-19 spread. In particular, considering the roadmap for lifting COVID-19 restrictions defined by the Italian Government, the model shows that if none of the infection-control measures is applied, the number of infected cases is bound to increase, leading to a second wave of infections. However, this can be substantially contained when infection-control measures are implemented. In particular, the model results highlight that the combinations of individual-level measures with undetected infection diagnosis can be effective to controlling the virus spread even when their singular implementation does not reach a high level of efficacy (e.g. 40% and 20%, respectively).

## Conclusion

Taken together, our results show that control measures have proven effective in containing the epidemic, neutralizing or at least limiting the potential dangerous impact of a large proportion of undetected cases. To the best of our knowledge, no other papers were proposed to study the COVID-19 outbreak considering different control strategies. Our model is an effective tool that can be used to model different scenarios. Moreover, we believe that our findings can help the policymakers in the enforcement of the best combination of infection-control measures potentially leading to the extinction of the COVID-19 epidemic.

## Supplementary information

**Additional file 1** S1 Introduction, S2 Mathematical Model, S3 Model fitting, S4 Parameters, S5 Results.

## Data Availability

All surveillance data, the SEIRS model and the parameters are available at https://github.com/qBioTurin/COVID-19. This article is present on a e&p repository website and can be accessed on https://repo.epiprev.it/index.php/2020/05/22/ impacts-of-reopening-strategies-for-covid-19-epidemic-a-modeling-study-in-piedmont-region/. This article is not published nor is under publication elsewhere

## References

[1] Carinci F (2020). Covid-19: Preparedness, Decentralisation, and the Hunt for Patient Zero. Br Med J.

[2] Department of Civil Protection I. COVID-19 ITALIA. http://opendatadpc.maps.arcgis.com/apps/opsdashboard/index.html/b0c68bce2cce478eaac82fe38d4138b1. Accessed 23 Mar 2020.

[3] Spina S, Marrazzo F, Migliari M, Stucchi R, Sforza A, Fumagalli R (2020). The response of Milan’s Emergency Medical System to the COVID-19 outbreak in Italy. Lancet.

[4] Holmdahl I, Buckee C (2020). Wrong but Useful - What Covid-19 Epidemiologic Models Can and Cannot Tell Us. N Engl J Med.

[5] Roosa K, Lee Y, Luo R, Kirpich A, Rothenberg R, Hyman J (2020). Real-time forecasts of the COVID-19 epidemic in China from February 5th to February 24th, 2020. Infect Dis Model.

[6] Moirano G, Richiardi L, Novara C, Maule M. Approaches to daily monitoring of the SARS-CoV-2 outbreak in Northern Italy. Fronteirs Public Health. 2020:1–10. in press. 10.3389/fpubh.2020.00222 PMC7256452 32574301

[7] Ferguson N, Laydon D, Nedjati-Gilani G, Imai N, Ainslie K, Baguelin M, Bhatia S, Boonyasiri A, Cucunubá Z, Cuomo-Dannenburg G, Dighe A. Impact of non-pharmaceutical interventions (NPIs) to reduce COVID-19 mortality and healthcare demand. Imperial College COVID-19 Response Team. 2020;:1–20.

[8] Kissler S, Tedijanto C, Goldstein E, Grad Y, Lipsitch M (2020). Projecting the transmission dynamics of SARS-CoV-2 through the postpandemic period. Science.

[9] Vollmer M, et al. Report 20: Using mobility to estimate the transmission intensity of COVID-19 in Italy: A subnational analysis with future scenarios. Imperial College COVID-19 Response Team. 2020;:1–17.

[10] Giordano G, Blanchini F, Bruno R, et al. Modelling the COVID-19 epidemic and implementation of population-wide interventions in Italy. 1–6. 2020. 10.1038/s41591-020-0883-7 PMC7175834 32322102

[11] Castagno P, Pernice S, Ghetti G, Povero M, Pradelli L, Paolotti D, Balbo G, Sereno M, Beccuti M (2020). A computational framework for modeling and studying pertussis epidemiology and vaccination. BMC Bioinformatics.

[12] Presidenza del Consiglio dei Ministri - Dipartimento della Protezione Civile. Italian survelliance data. https://github.com/pcm-dpc/COVID-19. Accessed: 28 Mar 2020.

[13] Prem K, Cook A, Jit M (2017). Projecting social contact matrices in 152 countries using contact surveys and demographic data. PLoS Comput Biol.

[14] Lin Q, Zhao S, Gao D, Lou Y, Yang S, Musa S, Wang M, Cai Y, Wang W, Yang L (2020). A conceptual model for the outbreak of Coronavirus disease 2019 (COVID-19) in Wuhan, China with individual reaction and governmental action. Int J Infect Dis.

[15] National Institute of Infectious Diseases J. Field Briefing: Diamond Princess COVID-19 Cases. https://www.niid.go.jp/niid/en/2019-ncov-e/9407-covid-dp-fe-01.html. Published: 2020-02-19.

[16] Trabucchi M, De Leo D (2020). Nursing homes or besieged castles: Covid-19 in northern italy. Lancet Psychiatry.

