# Supplementary material for: Impacts of reopening strategies for COVID-19 epidemic: a modeling study in Piedmont region
Source: BMC Infect Dis. 2020 Oct 28;20:798. doi: 10.1186/s12879-020-05490-w (PMC7592194; doi:10.1186/s12879-020-05490-w)
Supplement: Supplementary file 1 — Additional file 1 S1 Introduction, S2 Mathematical Model, S3 Model fitting, S4 Parameters, S5 Results. [file 12879_2020_5490_MOESM1_ESM.pdf]

Impacts of Reopening Strategies for COVID-19 Epidemic: A Modeling Study in Piedmont Region

Simone Pernice[1†], Paolo Castagno[1†], Linda Marcotulli[1], Milena Maria Maule[2], Lorenzo Richiardi[2], Giovenale Moirano[2], Matteo Sereno[1*^], Francesca Cordero[1^] and Marco Beccuti[1^]

[*]Correspondence: matteo.sereno@unito.it
[1]Department of Computer Science, University of Torino, Corso Svizzera 185, 10149, Torino, Italy
Full list of author information is available at the end of the article
[†]These authors contributed equally to this work. [^]These authors jointly supervised this work.

S1 Introduction

The case study of our model is the Piedmont region, one of the most affected northern Italian region. Before the re-opening phases, on May 2nd the total cases was 26.039. The Figure S1 A reports the distribution of the total cases among the Piedmont' provinces. In Figure S1 B is reported the distribution of the cases between the age groups following the proportion reported in Table S2.

S2 Mathematical Model

In this work, we extended the SEIRS model in order to cope with both the Sars-CoV-2 diffusion peculiarities and the effects of the policy makers decision on the population. Firstly, exposed (E) subjects when move to their infectious period are divided in *undetected infected* (I_u), *quarantined infected* (I_q) –i.e., individuals showing mild to moderate symptoms– and *hospitalized infected* (I_h) –i.e., individuals with severe symptoms. In this work, we do not address the evolution of the disease (e.g., transitions between I_q and I_h are not modelled), except for patients in the I_h compartment whose condition may get worse to the death.
One key aspect of the diffusion dynamic of Sars-CoV-2 disease, that is still largely unknown, is the real amount of infected individuals in the population. Indeed, it is largely agreed the presence of undetected infects in the population, either they are asymptomatic or just showing mild symptoms. Nonetheless, such undetected infects represent a key unknown variable in controlling the pandemic after gradually lifting COVID-19 restrictions. To study the effect of implementing contact tracing and swab testing, we added in the model the transition from compartment I_u toward I_q modelling the detection of the undetected infects.
Furthermore, the population is structured in three age sub-classes $\mathcal{A} = \{a_i |\ a_1 \in (0 \sim 19], a_2 \in [20 \sim 69], a_3 \in 70^+\}$. Within each age class, individuals are homogeneous both for their usual social activities and for the susceptibility to the disease. Hence, this layered population enables the model to track age specific incidence of the disease and the age specific social activity patterns. Specifically, we consider four different contact types, as reported in [1]: within the family unit $(Home)$, at school $(School)$, at work $(Work)$ and other contacts $(Other)$. Clearly, direct contacts are the main driver in the diffusion of the pathogen, while the quarantine regimes (η), the actions undertaken by the policy-makers (α) and the adoption of Personal Protective Equipment (p) are targeted to reduce them.
We report here the compartmental model formulation:

$$
\begin{cases}
\frac{dS_i}{dt} &= -\sum_{j=1}^{|\mathcal{A}|} \iota_i S_i \frac{(B(i,j,t,u)I_{uj} + B(i,j,t,q)I_{qj} + B(i,j,t,h)I_{hj})}{N_j} + \nu R_i \\
\frac{dE_i}{dt} &= \sum_{j=1}^{|\mathcal{A}|} \iota_i S_i \frac{(B(i,j,t,u)I_{uj} + B(i,j,t,q)I_{qj} + B(i,j,t,h)I_{hj})}{N_j} - (\lambda_u + \lambda_q + \lambda_h) E_i \\
\frac{dI_{ui}}{dt} &= \lambda_u E_i - \rho I_{ui} - \theta(i,t) I_{ui} \\
\frac{dI_{qi}}{dt} &= \lambda_q E_i - \rho I_{qi} + \theta(i,t) I_{ui} \\
\frac{dI_{hi}}{dt} &= \lambda_h E_i - (\sigma_i + \rho) I_{hi} \\
\frac{dR_i}{dt} &= \rho I_{ui} + \rho I_{qi} + \rho I_{hi} \\
\frac{dD_i}{dt} &= \sigma_i I_{hi} \\
\frac{dP}{dt} &= \sum_{i=1}^{|\mathcal{A}|} (\sigma_i I_{hi} + \lambda_h E_i) - \mu P \\
N_i &= S_i + E_i + I_{ui} + I_{qi} + I_{hi} + R_i,
\end{cases}
\tag{1}
$$

where $B(i,j,t,s)$ represents the **force of infection (FOI)** and it is defined as a time t, age class i, j, and infected categories s dependent function

$$
B(i,j,t,s) = \omega(t)\ p(t) \sum_{c \in \mathbf{C}} \alpha(t,c)\ \eta_{s,c}\ \beta^c_{i,j}, \qquad i,j \in \mathcal{A} \wedge s \in \mathcal{S}. \tag{2}
$$

Figure S1 (A) The total infected cases distributed in the counties of the Piedmont region.The intense of the color indicates higher propensity of making the contact. (B) The distribution of infected cases in the three age groups

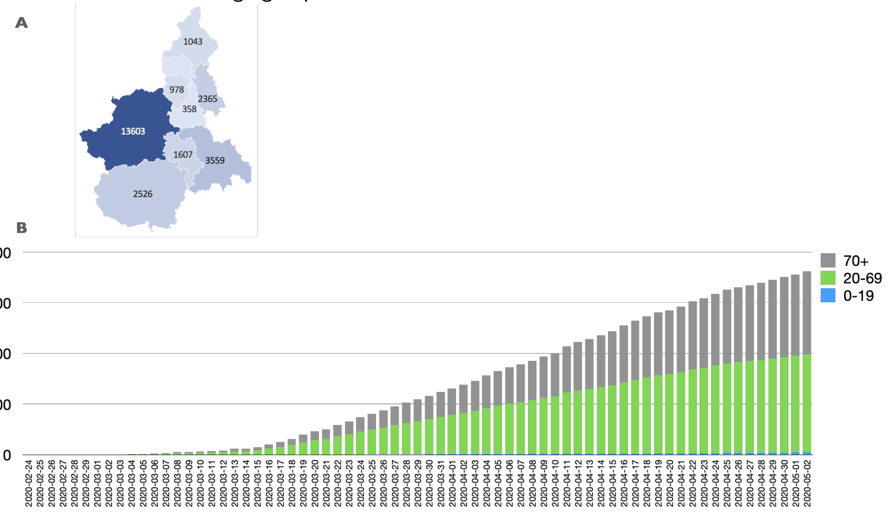

In details,

- $\mathcal{A}$, $\mathcal{S}$ represent the set of age classes and infected categories, respectively;
- $\alpha(t, c)$ is the strength of governmental action, defined as a step function depending on the time period;
- $\eta_{s,c}$ is the amount of social interaction considering the contact category $c \in \mathbf{C}$ of the infectious sub-class $s \in \mathcal{S}$;
- $\beta^{c}_{i,j}$ is a constant **contact rate**, depending on (1) the age classes of both the susceptible (i) and the infected (j) individuals, and (2) the contact categories [1];
- $p(t)$ describes the comply the Personal Protective Equipment (PPE) and social distancing measures imposed by the Italian government;
- $\omega(t)$ represents the intensity of the population response given the disease severity (k) with respect to the number of deaths and hospitalized infected individuals by CODIV-19 (P) in the last 40 days, assuming that the higher is P, the more prone to reduce the contacts is the population [2]. This is defined as follow:

$$\omega\,(t) = \left(1 - \frac{P(t)}{\sum_{i=1}^{|\mathcal{A}|} N_i}\right)^{k}. \tag{3}$$

Furthermore, Table S1 provides a detailed description of the parameter used in the compartmental model and further provides the values of those known parameters together with the reference to the original contribution.

S3 Model fitting

In the model presented in Equation 1 there are 13 parameters that are characterized by a high uncertainty, due to the recent onset of the disease and their difficulty of being empirically measured. Hence, the first step in our analysis consist of calibrating the set of these parameters according to the COVID-19 surveillance data available for the Piedmont region. Specifically, we adopt a best fitting trajectory to approximate the parameters which minimizes the mean squares error calculated with respect to the number of infected cases and deaths reported in [6]. Due the great number of parameters with high uncertainty, in addition to well reproduce the available data, their estimation is further controlled by the reproduction number R_0, calculated by exploiting the Next Generation Matrix method [7].

Nonetheless, the available information in [6] only provides an aggregated overview of the number quarantine infected individuals with mild symptoms, the number of hospitalized infected individuals with moderate or severe symptom and the number of deaths, without taking into account any age subdivision. Therefore, in order to provide a realistic distribution of cases in the population classes defined as in $\mathcal{A}$, the number of real cases has been distributed according to the disease incidence reported in Table S2 for the Piedmont region.

Table S1 Notation

Symbol	Value	Description	
$\mathcal{A}$	$\{1, \ldots, 3\}$	Set of age classes. Each age class corresponds to one of the following age ranges: 0-19, 20-69, and 70++	Assumption
$\mathcal{S}$	$\{u, q, h\}$	Set of infected categories. u: undetected infected individuals; q: quarantine infected individuals with mild symptoms; h: hospitalized infected individuals with moderate or severe symptoms	Assumption
$\mathbf{C}$	$\{Home,\ Work,\ School,\ Other\}$	Set of contact categories.	[1]
$\eta_{s,c},\ s \in \mathcal{S},\ c \in \mathbf{C}$	see Table S5	Amount of social interaction of the infected category s	Assumption
$\lambda_s, s \in \mathcal{S}$	$0.2\, r_s$	Rates at which an exposed individual becomes an infected individual in sub-class s. It is computed as the inverse of the incubation period time (5 days) multiplied by the ratio (r_s) of new infected in category s (see Table S4).	[3]
$\beta^c_{i,j},\ i, j \in \mathcal{A},\ c \in \mathbf{C}$		Contact rate between a susceptible of age class i and an infect of class j, considering the contact category c.	[1]
$\iota_i,\ i \in \mathcal{A}$		Age dependent probability of infection given a contact.	Estimated
ρ	0.2	The reciprocal of the mean infectious period (5 days). The time of infectiousness was derived from empirical estimates of the serial interval, the time between successive cases in a chain of transmission.	[4]
$\sigma_i,\ i \in \mathcal{A}$		Death rate for patients with sever symptoms in age class i.	Estimated
$\alpha(t, c),\ c \in \mathbf{C}$	see Table S6	Governmental action strength at time t	Estimated
k		Intensity of the population response w.r.t the COVID-19 perceived hazard	Estimated
$\theta(i, t),\ i \in \mathcal{A}$		Age and time dependent detection rate with which an undetected infected individual is discovered becoming a quarantine infected individuals	Estimated/Assumption
μ	0.025	Mean duration of public reaction (40 days)	Assumption
ν	0.001826	The reciprocal of the mean immunization period of the recovered individuals, equal to 1.5 years. This is an average value computed considering the immunity values of SARS and MERS	[5]

Table S2 Incidence of COVID-19 infections and deaths in Piedmont

	Updated at	Age classes $0-19$	$20-69$	$70++$	Reference
percentage of infection	2020-03-17	1%	60.1%	38.9%	[8]
	2020-03-22	0.8%	61.7%	37.5%	[9]
	2020-04-16	0.89%	55.1%	44.01%	[10]
	2020-04-26	1.3%	53.55%	45.15%	[11]
percentage of death	2020-04-15	0%	16.5%	83.5%	[12]

To fit the surveillance data with our model, we performed the calibration with the deterministic model of Equation 1 on Intel Xeon processor @ 2GHz. In particular a global optimization algorithm, based on *generalized simulated annealing method* [13], was exploited to estimate the 13 parameters with high uncertainty. In details, three parameters represent the probability of infection for each age class, four parameters reflect the governmental action strength at time epoch t (i.e., $\alpha(t)$ with $t \in \{$ March 8th, March 21$^{\text{st}}\}$), one parameter describes the intensity of the population response (i.e., k), two parameters represent the death rate for the hospitalized patients (i.e., $\sigma_i,\ i = 2,\ 3$, fixing $\sigma_1 = 0$), two parameters are the initial condition for the undetected and quarantine infected individuals, and the remainder parameter represents the detection rate for the third age class starting from the 1st April. The estimated values are reported in Table S3, and the basic reproduction number resulting from such parameters is $R_0 = 2.7$ considering the initial stage of the pandemic in Piedmont the period from February 21$^{\text{st}}\}$ to February 25$^{\text{st}}\}$.

Therefore, the model calibration was carried out considering the proportion between undetected and detected infected individuals (i.e., given by the sum of the quarantined and hospitalized infected individuals) to be one-to-one on average as reported in [14]. Neglecting the fraction of undetected, the remaining portion of the cases is divided into Ih and Iq as reported in [6]. The number of hospitalised patients, which is in average on the time interval from 24th February to the 4th May the 45% of the total reported cases, is considered for simplicity equal for each age class.

According to [15], we assumed that the initial system state is $S_1 = 733130,\ S_2 = 2780600,\ S_3 = 842676,$; while the initial number of infected individuals is estimated as $Iq_2 = 4,\ Iu_1 + Iu_2 + Iu_3 = 100$, where the undetected individuals were distributed proportionally to the age class population size. All the other compartments are set to zero.

Table S3 Estimated parameters of the best fitting trajectory.

Parameter	Value			
	i =	1	2	3
ι_i		0.0095	0.08	0.285
σ_i		-	0.019	0.33
$\alpha(t, c)$	c = / t =	$Work$	$Other$	
	March 8th	0.75	0.65	
	March 21st	0.4	0.3	
k	60			
Iq_2	4			
$Iu_1 + Iu_2 + Iu_3$	100			
θ (3, April 1st)	0.12			

S4 Parameters

Table S4 Ratio r_s of new infected in category s (u, q and h).

Scenario	u	q	h
One-to-one	0.50	0.275	0.225

Table S5 Contact restriction due to the infection.

Infect category	$Home$	$Work$	$School$	$Other$
u	1	1	1	1
q	0.1	0	0	0
h	0	0	0	0.05

Table S6 Governmental actions strength for each contact category characterized by three restrictions (first three rows) and three releases (last three rows).

Date	$Home$	$Work$	$School$	$Other$
February 25th	1	1	0	1
March 8th	1	0.75	0	0.65
March 21st	1	0.4	0	0.3
May 4th	1	0.5	0	0.35
May 18th	1	0.6	0	0.4
June 1st	1	0.9	0	0.5

S4.1 Contact Matrix

We exploited the Italian synthetic age-specific contact matrix estimated in [1]. In particular, this matrix is defined as a linear combination of four matrices depending on the location of contact, i.e, $Home$, $School$, $Work$, and $Other$. Since our model is defined for three age classes (0-19, 20-69 and 70++), while in [1] the age classes are defined as 5-year bands until age 70 years and a single category aged 75++ (resulting in 16 age categories), we have to scale the rates proportionally to the size and number of the new age classes. In Fig. S5 the age-specific and location-specific contact matrices are reported. In details, the columns represent the location of contact, while the rows report the seven phases in the simulation window: the usual contact rates, namely *Normal*, the three public restrictions, and the three re-opening phases. The intense of the color indicates higher propensity of making the contact considering a specific phase.

S5 Results

Figures S2 and S3 show the time evolution of infected individuals and the deaths due to Sars-CoV-2 derived by the model considering the optimal parameter values estimated in the calibration phase. In details, the stacked bar charts in Figure S2d) shows the proportion of infected individuals in the population. Comparing this surveillance data with those obtained by our calibrated model (i.e, given by the sum of the light blue and blue-gray bars in Fig.S2) we can clearly point out that a good level of accordance is achieved. Similar conclusion can be derived when we focus on the deaths due to Sars-CoV-2. Indeed Fig.S3 d) shows certainly that the calibrated model is able to consistently mimic the measured death cases (i.e., red line) too, for both if we look at the aggregated information and if we compare single age classes. Furthermore, Figures S2 from a) to c) provide a detailed overview on how the infects spread across the different age classes.
Figure S4 shows 5000 different stochastic realization of our mechanistic model with the corresponding median trend and interquartile range. It can be noted that in the initial stages of the pandemic the stochastic behaviour of our model foresees a slightly higher number of infected cases, but starting from March 21st the Surveillance data (red line) lies within the interquaritle range, depicted as a light-green shaded area.

Figure S2 Model fitting of the surveillance data. Histogram's bars show the number of infected individuals for each age class (a) Age class $0-19$; b) Age class $20-69$; c) Age class $70++$; d) Overall population) with severe symptoms I_h, under quarantine restriction in their houses I_q and undetected infects I_u. The red curve represents the surveillance data, which does not account for undetected cases.

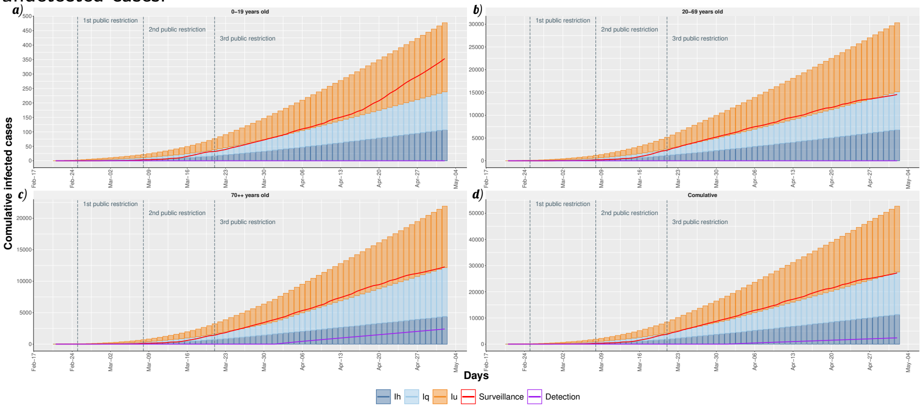

Author details

[1]Department of Computer Science, University of Torino, Corso Svizzera 185, 10149, Torino, Italy. [2]Cancer Epidemiology Unit, Department of Medical Sciences, University of Torino - CPO Piemonte, Via Santena 7, 10126, Torino, Italy.

References

1. Prem, K., Cook, A.R., Jit, M.: Projecting social contact matrices in 152 countries using contact surveys and demographic data. PLoS computational biology **13**(9), 1005697 (2017)

Figure S3 Model fitting of the surveillance data considering the number of deaths for each age class (a) Age class $20-69$; b) Age class $70++$; c) Overall population). The age class $0-19$ is not showed because no deaths are reported in Piedmont region. The red curve represents the surveillance data, which does not account for undetected cases.

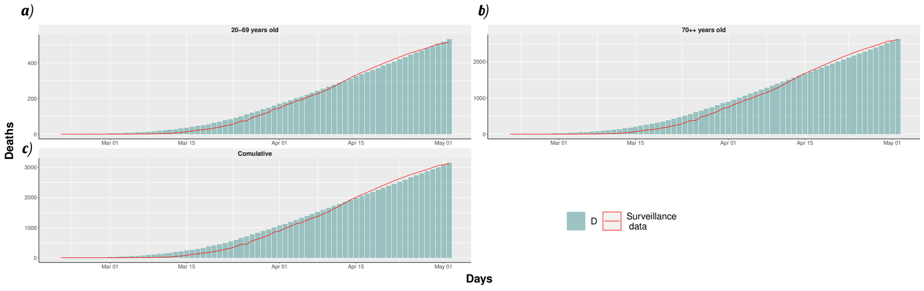

Figure S4 Stochastic simulations of the one-to-one ratio scenario. 5000 stochastic traces spanning the time period from February 21th to May 1st. The light-green shaded area shows the range containing traces within the 25th to 75th quantiles. The dark-green line is the median of all the traces and the red line is the surveillance. Vertical dashed lines mark the division between two time epoch.

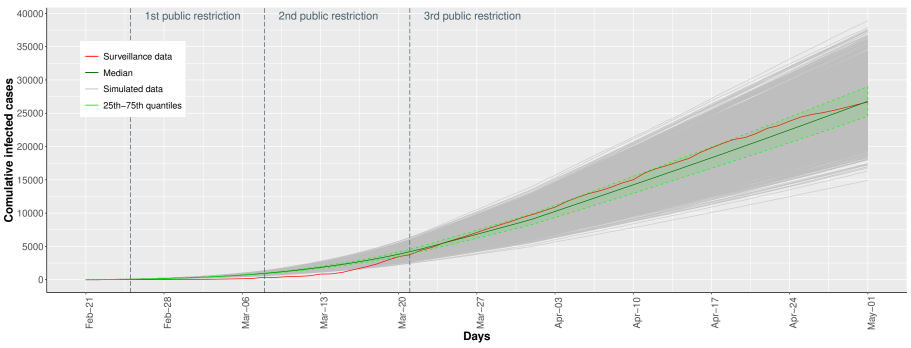

2. Lin, Q., Zhao, S., Gao, D., Lou, Y., Yang, S., Musa, S.S., Wang, M.H., Cai, Y., Wang, W., Yang, L., *et al.*: A conceptual model for the outbreak of coronavirus disease 2019 (covid-19) in wuhan, china with individual reaction and governmental action. International Journal of Infectious Diseases **93**, 211–216 (2020)
3. Lauer, S.A., Grantz, K.H., Bi, Q., Jones, F.K., Zheng, Q., Meredith, H.R., Azman, A.S., Reich, N.G., Lessler, J.: The Incubation Period of Coronavirus Disease 2019 (COVID-19) From Publicly Reported Confirmed Cases: Estimation and Application. Annals of Internal Medicine (2020)
4. Nishiura, H., Linton, N.M., Akhmetzhanov, A.R.: Serial interval of novel coronavirus (2019-ncov) infections. medRxiv (2020). doi:10.1101/2020.02.03.20019497
5. Wu, L., Wang, N., Chang, Y., et al.: Duration of antibody responses after severe acute respiratory syndrome. Emerging Infectious Diseases **13(10)**, 1562–1564 (2007)
6. Presidenza del Consiglio dei Ministri - Dipartimento della Protezione Civile: Italian surveillance data. `https://github.com/pcm-dpc/COVID-19`. Accessed: 2020-03-28
7. Van den Driessche, P., Watmough, J.: Reproduction numbers and sub-threshold endemic equilibria for compartmental models of disease transmission. Mathematical biosciences **180**(1-2), 29–48 (2002)
8. Di Pietrantonj, C., Serale, M., Ferrara, L., Tiberti, D., Lombardi, D., Raso, R., Finesso, A., Pellegrino, D., Pasqualini, C.: Epidemia COVID-19 Regione Piemonte: REPORT DEL 22 MARZO 2020. `https://www.seremi.it/`. Version: 2020-03-17
9. Di Pietrantonj, C., Serale, M., Ferrara, L., Tiberti, D., Lombardi, D., Raso, R., Finesso, A., Pellegrino, D., Pasqualini, C.: Epidemia COVID-19 Regione Piemonte: REPORT DEL 22 MARZO 2020. `https://www.seremi.it/`. Version: 2020-03-22
10. Di Pietrantonj, C.: Epidemia COVID-19 Regione Piemonte: situazione al 16.04.2020 ore 10:00. `https://www.seremi.it/`. Version: 2020-04-16
11. Di Pietrantonj, C.: Epidemia COVID-19 Regione Piemonte: situazione al 26.04.2020 ore 10:00. `https://www.seremi.it/`. Version: 2020-04-26
12. Task force COVID-19: Sorveglianza Integrata COVID-19 in Italia. Istituto Superiore di Sanitá - Dipartimento

	Home			Other			Work			School			
	0-19	20-70	>70	0-19	20-70	>70	0-19	20-70	>70	0-19	20-70	>70	
0-19	1.94626091	1.85788621	0.05751326	3.59022198	2.65505839	0.19290783	0.01111344	0.0120437	6.56E-06	5.18483237	0.68061173	0.0001179	Normal
20-70	0.66185773	2.150229	0.06691802	0.68828975	6.14448455	0.36851142	0.21203735	3.74408731	2.60E-05	1.22937062	0.36937897	0.00087144	
>70	0.43331945	1.24103351	0.45030494	0.14779459	4.41545631	0.44487167	2.76E-06	0.00022143	1.24E-05	1.90E-64	4.74E-66	6.75E-92	

	Home			Other			Work			School			
	0-19	20-70	>70	0-19	20-70	>70	0-19	20-70	>70	0-19	20-70	>70	
0-19	1.94626091	1.85788621	0.05751326	3.59022198	2.65505839	0.19290783	0.00111134	0.00120437	6.56E-07	0	0	0	First restriction
20-70	0.66185773	2.150229	0.06691802	0.68828975	6.14448455	0.36851142	0.19083362	3.36967858	2.34E-05	0	0	0	
>70	0.43331945	1.24103351	0.45030494	0.14779459	4.41545631	0.44487167	2.48E-06	0.00019929	1.11E-05	0	0	0	

	Home			Other			Work			School			
	0-19	20-70	>70	0-19	20-70	>70	0-19	20-70	>70	0-19	20-70	>70	
0-19	1.94626091	1.85788621	0.05751326	2.33364429	1.72578795	0.12539009	0.00833508	0.00903278	4.92E-06	0	0	0	Second restriction
20-70	0.66185773	2.150229	0.06691802	0.44738834	3.99391496	0.23953242	0.15902801	2.80806548	1.95E-05	0	0	0	
>70	0.43331945	1.24103351	0.45030494	0.09606649	2.8700466	0.28916658	2.07E-06	0.00016607	9.29E-06	0	0	0	

	Home			Other			Work			School			
	0-19	20-70	>70	0-19	20-70	>70	0-19	20-70	>70	0-19	20-70	>70	
0-19	1.94626091	1.85788621	0.05751326	1.07706659	0.79651752	0.05787235	0.00444537	0.00481748	2.62E-06	0	0	0	Thrid restriction
20-70	0.66185773	2.150229	0.06691802	0.20648692	1.84334537	0.11055342	0.08481494	1.49763492	1.04E-05	0	0	0	
>70	0.43331945	1.24103351	0.45030494	0.04433838	1.32463689	0.1334615	1.10E-06	8.86E-05	4.95E-06	0	0	0	

	Home			Other			Work			School			
	0-19	20-70	>70	0-19	20-70	>70	0-19	20-70	>70	0-19	20-70	>70	
0-19	1.94626091	1.85788621	0.05751326	1.25657769	0.92927044	0.06751774	0.00555672	0.00602185	3.28E-06	0	0	0	First reopening phase
20-70	0.66185773	2.150229	0.06691802	0.24090141	2.15056959	0.128979	0.10601868	1.87204365	1.30E-05	0	0	0	
>70	0.43331945	1.24103351	0.45030494	0.05172811	1.54540971	0.15570508	1.38E-06	0.00011072	6.19E-06	0	0	0	

	Home			Other			Work			School			
	0-19	20-70	>70	0-19	20-70	>70	0-19	20-70	>70	0-19	20-70	>70	
0-19	1.94626091	1.85788621	0.05751326	1.43608879	1.06202336	0.07716313	0.00666806	0.00722622	3.94E-06	0	0	0	Second reopening phase
20-70	0.66185773	2.150229	0.06691802	0.2753159	2.45779382	0.14740457	0.12722241	2.24645238	1.56E-05	0	0	0	
>70	0.43331945	1.24103351	0.45030494	0.05911784	1.76618252	0.17794867	1.65E-06	0.00013286	7.43E-06	0	0	0	

	Home			Other			Work			School			
	0-19	20-70	>70	0-19	20-70	>70	0-19	20-70	>70	0-19	20-70	>70	
0-19	1.94626091	1.85788621	0.05751326	1.79511099	1.32752919	0.09645391	0.01000209	0.01083933	5.91E-06	0	0	0	Third reopening phase
20-70	0.66185773	2.150229	0.06691802	0.34414487	3.07224228	0.18425571	0.19083362	3.36967858	2.34E-05	0	0	0	
>70	0.43331945	1.24103351	0.45030494	0.0738973	2.20772816	0.22243583	2.48E-06	0.00019929	1.11E-05	0	0	0	

Figure S5 The age-specific and location-specific (columns) contact matrices are reported for each phase (rows) in the simulation window. The intense of the color indicates higher propensity of making the contact.

Malattie Infettive e Servizio di Informatica, Version: 2020-04-15, https://www.epicentro.iss.it/coronavirus/bollettino/Infografica_15aprile\%20ITA.pdf

13. Yang Xiang, Gubian, S., Suomela, B., Hoeng, J.: Generalized simulated annealing for efficient global optimization: the GenSA package for R. The R Journal (2012). Forthcoming
14. National Institute of Infectious Diseases, J.: Field Briefing: Diamond Princess COVID-19 Cases. https://www.niid.go.jp/niid/en/2019-ncov-e/9407-covid-dp-fe-01.html. Published: 2020-02-19
15. Ministero della Salute. Resident population on 1st January in Piedmont. http://dati.istat.it/Index.aspx?QueryId=18540
